# The Evaluation of Bioelectrical Activity of Pelvic Floor Muscles Depending on Probe Location: A Pilot Study

**DOI:** 10.1155/2013/238312

**Published:** 2013-12-11

**Authors:** Tomasz Halski, Kuba Ptaszkowski, Lucyna Słupska, Robert Dymarek

**Affiliations:** ^1^Department of Physiotherapy, Public Higher Medical Professional School in Opole, Katowicka 68, 45-060 Opole, Poland; ^2^Department of Obstetrics, Faculty of Health Science, Wroclaw Medical University, K. Bartla 5, 51-618 Wroclaw, Poland; ^3^Department of Clinical Biomechanics and Physiotherapy in Motor System Disorders, Faculty of Health Science, Wroclaw Medical University, Grunwaldzka 2, 50-355 Wroclaw, Poland; ^4^Department of Nervous System Diseases, Faculty of Health Science, Wroclaw Medical University, K. Bartla 5, 51-618 Wroclaw, Poland

## Abstract

*Objectives*. The main objective was to determine how the depth of probe placement affects functional and resting bioelectrical activity of the PFM and whether the recorded signal might be dependent on the direction in which the probe is rotated. *Participants*. The study comprised of healthy, nulliparous women between the ages of 21 and 25. *Outcome Measures*. Bioelectric activity of the PFM was recorded from four locations of the vagina by surface EMG and vaginal probe. *Results*. There were no statistically significant differences between the results during functional sEMG activity. During resting sEMG activity, the highest bioelectrical activity of the PFM was observed in the L1 and the lowest in the L4 and a statistically significant difference between the highest and the lowest results of resting sEMG activity was observed (*P* = 0.0043). *Conclusion*. Different electrodes placement during functional contraction of PFM does not affect the obtained results in sEMG evaluation. In order to diagnose the highest resting activity of PFM the recording plates should be placed toward the anterior vaginal wall and distally from the introitus. However, all of the PFM have similar bioelectrical activity and it seems that these muscles could be treated as a single muscle.

## 1. Introduction

A proper assessment of the pelvic floor muscles (PFM) is an important part in the diagnosis and treatment associated with pelvic floor dysfunction, particularly with respect to urinary incontinence, faecal incontinence, or genital prolapse in women [1–9]. Methods for evaluating the strength and the endurance of the PFM are subjective transvaginal digital palpation (e.g., The Oxford Scale or The Modified Oxford Scale) and objective methods such as perineometry and electromyography (EMG) are often indicated [1, 10–18]. In understanding the proper neural control as well as normal and pathological activity of the PFM a needle or surface EMG is proving to be a useful tool [19]. Increasingly common apparatus for the objective assessment of PFM is surface electromyography (sEMG) with a vaginal probe [20–23]. Some studies [24–27] indicate that exact assessment of PFM function with the probe is facilitated by the fact that these muscles can behave as a single muscle during resting and functional activity. However, in accordance with the principles of evidence-based medicine we should seek to standardize measurements in terms of research equipment parameters, time, location of the measurement, and a patients position during the examination [28–35]. Reliable and consistent recording of PFM activity can be difficult, which transpires from the diversity of vaginal probes and their placement [36]. It is known that the shape and size of the probes may influence the results obtained, so it is important to optimize the type of the probe which is used to assess the strength of the PFM [19, 37].

## 2. Objectives

The wide differentiation in vaginal probes and the lack of clear methodology of their application prompted the authors to perform an evaluation of PFM bioelectrical activity corresponding to probe location. The main objective was to determine how the depth of probe placement affects the functional and resting bioelectrical activity of the PFM and whether the recorded signal might be dependent on the direction in which the probe is rotated. The probe was placed in two different orientations, toward the anterior or posterior wall of the vagina. A secondary objective was to evaluate any correlation between sEMG activities of the PFM which were measured at various areas of the vagina.

## 3. Materials and Methods

### 3.1. Subjects

**Figure 1:**
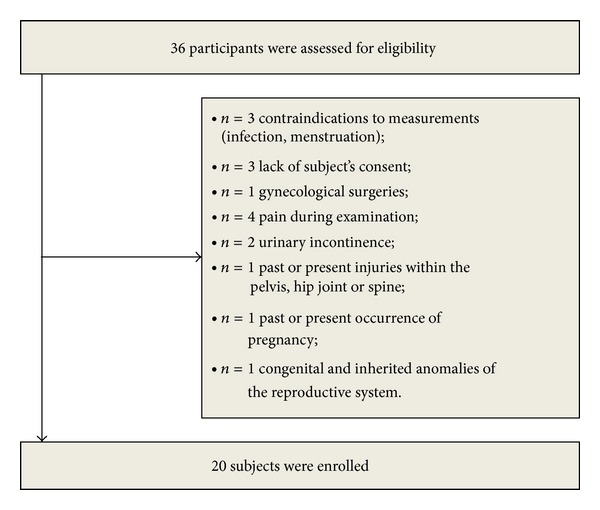
Flow diagram includes detailed information on the excluded participants.

This study was approved by the Bioethics Committee of the Wroclaw Medical University (KB-611/2012, Wroclaw, Poland) and all subjects provided written informed consent. Thirty-six healthy, nulliparous women were recruited from the Public Higher Medical Professional School population to participate. Women with a history of incontinence, gynaecological surgeries, congenital and inherited anomalies of the reproductive system, past or present injuries within the pelvis, hip joint or spine, and pregnancy were excluded, as well as women with contraindications to measurements (such as infection and menstruation) (Figure 1). Finally, the study comprised of twenty volunteers between the ages of 21 and 25 ($\overset{-}{x}=22.3$ years, SD = 1.28 years).

### 3.2. Electromyography

The electromyographic signal was registered by a dual-channel sEMG NeuroTrac ETS device integrated with computer software for digital analysis and report creation (Verity Medical Ltd., UK). This device is characterized by an amplitude range of 0.2–2000 *μ*V RMS continuous in the frequency band of 2–100 Hz and pulse width from 50 to 450 *μ*S for recording signals generated by muscles. Device sensitivity is established at a level 0.1 *μ*V (4% accuracy; readings ±0.3 mV at 200 Hz), with selectable bandpass filter (3 db bandwidth) and 50 Hz notch filter (33 dbs; 0.1% accuracy). The analogue signal recorded by the sEMG electrodes was amplified, filtered, and subsequently transformed into a digital signal. Such signal facilitated statistical analysis of acquired results and allowed for data representation in a graphical form. Mean values of muscle bioelectrical activity were given according to root mean square algorithm (RMS) [28, 38–40]. The monopolar, self-adhesive reference electrode was placed on the anterior superior iliac spine.

### 3.3. Probe Descriptions

To investigate the pelvic floor muscle activity we used Vaginal Probe Periprobe Optima 3 (Sugar International, France) with 3 independent, hemispherical, nickel-free electrodes (recording plates). The top (electrode A), the middle (electrode B), and the bottom (electrode C) electrodes of the probe are three detection surfaces. The probe has a total length of 12 cm and total weight of 21 g. The circumference of the top electrode is 7.5 cm. The circumference of the middle and the bottom electrode is 6.5 cm. The distance between the electrodes is 3.3 cm. The position of the probe was determined by a mark to be placed in line with the introitus of the vagina. The middle of electrode A is 8.7 cm from the introitus, the middle of electrode B is 5.4 cm from the introitus, and the middle of electrode C is 2.1 cm from the introitus.

### 3.4. Experimental Protocol

**Figure 2:**
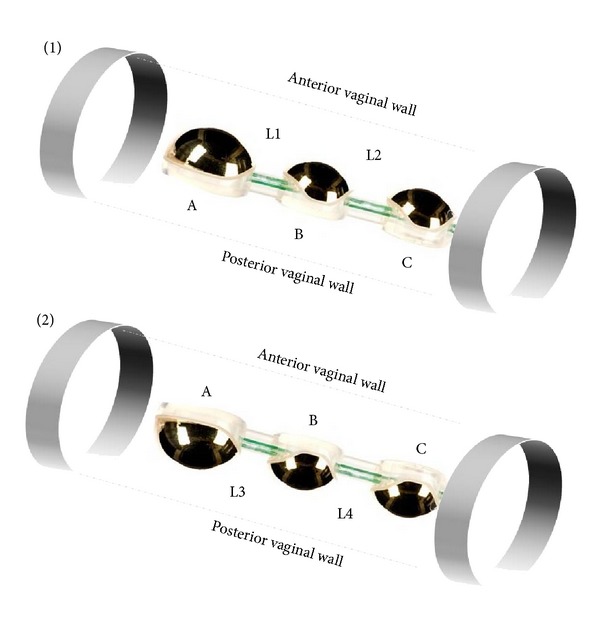
Location of the probe during measuring of PFM activity. (1) The probe was toward the anterior wall of the vagina. (2) The probe was toward the posterior wall of the vagina; L1, L2, L3, and L4: locations 1, 2, 3, and 4; A: the top electrode, B: the middle electrode, and C: the bottom electrode.

Measurement of electrical activity of the PFM was assessed in a standing position. Prior to measurements, each participant was instructed how to perform an isolated PFM contraction. Resting and functional sEMG activity (in microvolts *μ*V) were recorded. All of the women participating in this study were asked to contract the PFM as hard as possible for five seconds (functional activity). The contractions were repeated five times with five-second break between each contraction (resting activity). The probe was placed in two different orientations and measurement was performed when the probe was toward the anterior wall of the vagina and afterward toward the posterior wall. Bioelectric activity of the PFM was recorded from four locations (Figure 2).

 Location 1 (L1) is the circuit between electrodes A and B towards anterior wall of the vagina.
 Location 2 (L2) is the circuit between electrodes B and C towards anterior wall of the vagina.
 Location 3 (L3) is the circuit between electrodes A and B towards posterior wall of the vagina.
 Location 4 (L4) is the circuit between electrodes B and C towards posterior wall of the vagina.

### 3.5. Statistical Analysis

Statistical analysis was performed using Statistica 10. Analysis of variance (ANOVA) of Kruskal-Wallis was used to examine the difference between the sEMG activity in each location. A value of *P* < 0.05 was considered statistically significant. The differences between measurements obtained during resting and during functional sEMG activity were compared. In addition, Spearman correlation was made to show the relationship between the variables.

## 4. Results

**Figure 3:**
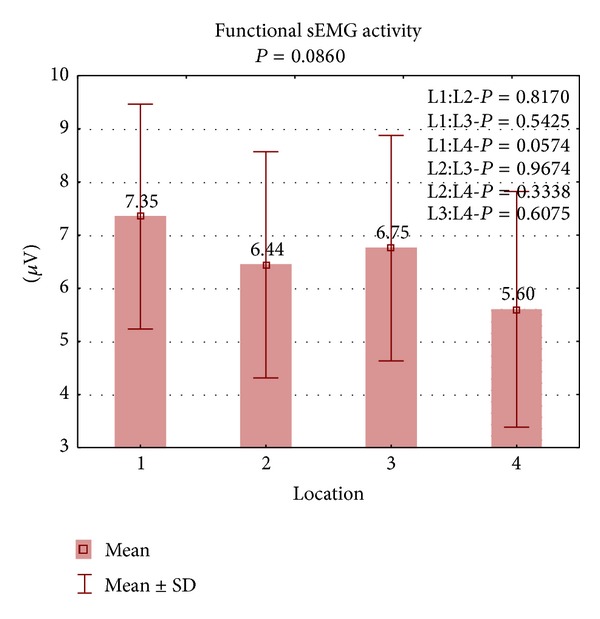
The results of PFM activity during functional sEMG activity in four locations.

**Figure 4:**
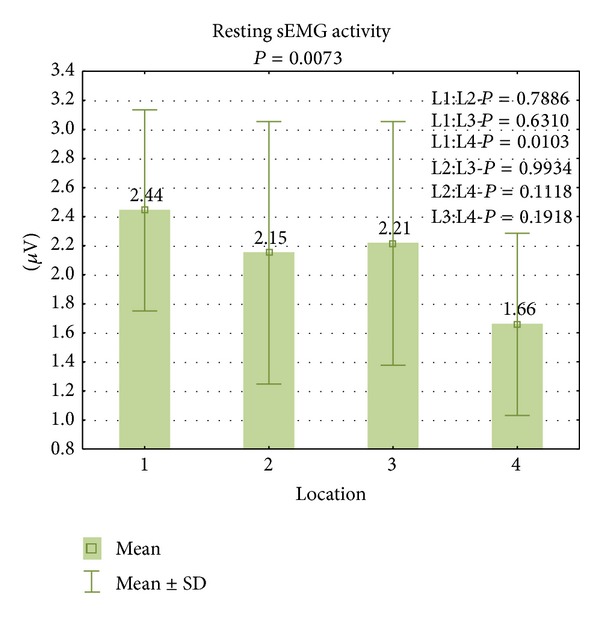
The results of PFM activity during resting sEMG activity in four locations.

There were no statistically significant differences between the results during functional sEMG activity (Figure 3). During resting sEMG activity, the highest bioelectrical activity of the PFM was observed in L1 ($\overset{-}{x}=2.4$ *μ*V, min-max: 1.3–4.0 *μ*V, SD = 0.69 *μ*V) and the lowest in the L4 ($\overset{-}{x}=1.7$ *μ*V, min-max: 0.9–3.2 *μ*V; SD = 0.63 *μ*V). A statistically significant difference between the highest and the lowest results of resting sEMG activity was observed (*P* = 0.0043) (Figure 4). Among other results, no statistically significant differences were registered.

In the study population, a statistically significant correlation was found for all analyzed variables. The correlations are presented in Tables 1 (for resting sEMG activity) and 2 (for functional sEMG activity). Spearman analysis showed statistical correlation between the results of PFM functional activity of four locations (Table 1): between L1 and L2: *P* = 0.0008, *r* = 0.69; L1 and L3: *P* = 0.0000, *r* = 0.85; L1 and L4: *P* = 0.0012, *r* = 0.67; L2 and L3: *P* = 0.0000, *r* = 0.83; L2 and L4: *P* = 0.0000, *r* = 0.89; L3 and L4: *P* = 0.0000, *r* = 0.87. We also observed statistical correlation between the results of PFM resting activity (Table 2): between L1 and L2: *P* = 0.0052, *r* = 0.60; L1 and L3: *P* = 0.0022, *r* = 0.64; L1 and L4: *P* = 0.0173, *r* = 0.53; L2 and L3: *P* = 0.0010, *r* = 0.68; L2 and L4: *P* = 0.0000, *r* = 0.81; L3 and L4: *P* = 0.0000, *r* = 0.83.

## 5. Discussion

This study tries to determine the evaluation of bioelectrical activity of the PFM according to probe location. The factors which were taken into consideration are depth of electrode placement and their orientation. Functional and resting sEMG activity was assessed with an Optima 3 vaginal probe.

The presented results pertain to the diagnostics of PFM activity and they confirm that the activity depends on the area where recording plates are located. Long probes have recording plates which can record the sEMG signal from areas of vaginal wall located distal to the vaginal introitus. Shorter probes collect the activity from proximal locations.

Voorham van der Zalm et al. [36] conducted similar assessment of the location of different types of electrodes. In their study they used five common probes which differed in shape, length, and width of the recording plates as well as in circumference, length of the probe, and place of insertion of the probe. The position of recording plates was evaluated in relation to puborectal muscles and examined by ultrasound. Although the study did not have a representative research group, on the basis of the results the authors recognize the value in conducting further studies in order to optimize the probes used.

Bø et al. [37] also noticed that the size and location of vaginal probes have an impact on the obtained results. In the assessment of the PFM they used two types of vaginal probes: the Camtech Squeez meter (length: 6.7 cm, diameter: 1.7 cm, location: the middle of the balloon was 3.5 cm from the introitus) and the Peritron (length: 10.8 cm, diameter: 2.8 cm, location: 0.5–1 cm of the probe was visible outside the introitus). The results of vaginal squeeze pressure varied depending on the type of vaginal probe used in the study. Therefore, the use of various electrodes in the studies does not allow for effective comparison of results.

A clinician should be able to match the appropriate type of probe depending on the therapeutic purpose (specifically in electrical stimulation). This is due to another feature of vaginal probes. In addition to their usage in the evaluation of PFM activity, they can be applied in the treatment of urinary incontinence. For example, in therapy for urge incontinence, the stimulation should include afferent nerve fibres of the plexus pelvicus and the pudendal nerve. In cases where the patient suffers from stress incontinence, electrical stimulation should influence the external sphincter and pelvic floor muscles. Thus, size, shape, length, width, and circumference play a significant role both in the diagnostics and in the therapy of urinary incontinence [36, 41, 42].

The distribution of forces acting on the vagina following pelvic floor contractions is varied which was confirmed by our results. Although, most of the results are not statistically significant, higher bioelectric activity was observed more distally from the introitus and on the anterior wall of the vagina. Constantinous and Omata's study [43] is another investigation into the distribution of forces acting on the vagina. They evaluated the distribution of anisotropic forces on the vagina following voluntary and reflex pelvic floor contractions. The probe with four pairs of force and displacement sensors was used to measure the pelvic floor closure force. The researchers observed significantly higher maximum forces of contraction in the anterior aspects of the vagina during reflex pelvic floor contractions. The unequal distribution of forces in the vaginal walls is the subject of other similar studies [44–47].

However Shafik's study [24] demonstrates that all of the pelvic floor muscles behave as one muscle since they contract or relax collectively, which was also noticed in this study in strong correlation between measurements from particular localizations. He explains this phenomenon by referring to the origin of pelvic floor muscles. External anal (EAS) and urethral sphincters (EUS) as well as the bulbocavernosus muscle (BC) arise from the puborectalis muscle (PR). Though the levator ani (pubococcygeus) is not descended from the puborectalis muscle, it shares with it its innervations through the pudendal nerve. Stimulation of sensory fibres of the pudendal nerve activates reflex contractions of the stimulated muscle and of all of the muscles supplied by this nerve. Nonetheless, he also confirmed a voluntary selective muscle activity and that each individual pelvic floor muscle can act independently of the others.

The results prompt for further studies, in order to find research tools for more accurate assessment of pelvic floor muscles, in addition to the evaluation by sEMG or perineometer. It may be very meaningful to diagnose the PFM using ultrasound or MRI. Furthermore, there is a need to strictly determine the methodology of measurements and the type of equipment used.

Attention should be given to the practical implications of this study. The clinical reliability and accuracy of PFM measurements are indeterminate and should be reevaluated. The study is highlighting aspects of the objectification of both the measurement and the measurement tools. In this pilot study the authors used a probe which has not yet been the subject of a randomized trial and which assessed the bioelectrical activity from various localizations of the vagina.

## 6. Limitation of the Study

Some limitations of the study were the small number of participants, no measurements in patients with pelvic floor dysfunction, and the lack of more sensitive multichannel sEMG. This study will be continued among patients with the pelvic floor dysfunction and complemented by measurements using different types of probes.

## 7. Conclusion

Different electrodes placement during functional contraction of the PFM does not affect the obtained results in sEMG evaluation. In order to diagnose the highest resting activity of the PFM the recording plates could be placed toward the anterior vaginal wall and distally from the introitus. However, all of the PFM have similar bioelectrical activity and it seems that these muscles could be treated as a single muscle. Therefore, it is appropriate to continue to conduct measurements of bioelectrical activity of the PFM, depending on the placement and the type of probes. Further experimental research should include a larger number of participants as well as individuals with lower urinary tract symptoms.

## Figures and Tables

**Table 1:** The correlation between the results of PFM activity of four locations—functional sEMG activity.

Functional sEMG activity of PFM				
	L1	L2	L3	L4
L1	—	*r* = 0.69 *P* = 0.0008	*r* = 0.85 *P* = 0.0000	*r* = 0.67 *P* = 0.0012
L2	*r* = 0.69 *P* = 0.0008	—	*r* = 0.83 *P* = 0.0000	*r* = 0.89 *P* = 0.0000
L3	*r* = 0.85 *P* = 0.0000	*r* = 0.83 *P* = 0.0000	—	*r* = 0.87 *P* = 0.0000
L4	*r* = 0.67 *P* = 0.0012	*r* = 0.89 *P* = 0.0000	*r* = 0.87 *P* = 0.0000	—

**Table 2:** The correlation between the results of PFM activity of four locations—resting sEMG activity.

Resting sEMG activity of PFM				
	L1	L2	L3	L4
L1	—	*r* = 0.60 *P* = 0.0052	*r* = 0.64 *P* = 0.0022	*r* = 0.53 *P* = 0.0173
L2	*r* = 0.60 *P* = 0.0052	—	*r* = 0.68 *P* = 0.0010	*r* = 0.81 *P* = 0.0000
L3	*r* = 0.64 *P* = 0.0022	*r* = 0.68 *P* = 0.0010	—	*r* = 0.83 *P* = 0.0000
L4	*r* = 0.53 *P* = 0.0173	*r* = 0.81 *P* = 0.0000	*r* = 0.83 *P* = 0.0000	—
